# Using the Listening2Faces App with Three Young Adults with Autism: A Feasibility Study

**DOI:** 10.1007/s41252-023-00390-x

**Published:** 2024-01-19

**Authors:** Alisa Baron, Vanessa Harwood, Cooper Woodard, Kaitlyn Anderson, Barbara Fernandes, Jessica Sullivan, Julia Irwin

**Affiliations:** 1 Department of Communicative Disorders, The University of Rhode Island, 25 W. Independence Way, Kingston, RI 02881 USA; 2 Groden Center, Providence, RI USA; 3 Smarty Ears Inc, Dallas, TX USA; 4 Department of Communication Sciences and Disorders, Hampton University, Hampton, VA USA; 5 Department of Psychology, Southern Connecticut State University, New Haven, CT USA

**Keywords:** Autism, Listening in noise, Application, Complex communication needs, Audiovisual speech perception

## Abstract

**Objectives:**

Listening2Faces (L2F) is a therapeutic, application-based training program designed to improve audiovisual speech perception for persons with communication disorders. The purpose of this research was to investigate the feasibility of using the L2F application with young adults with autism and complex communication needs.

**Methods:**

Three young adults with autism and complex communication needs completed baseline assessments and participated in training sessions within the L2F application. Behavioral supports, including the use of cognitive picture rehearsal, were used to support engagement with the L2F application. Descriptive statistics were used to provide (1) an overview of the level of participation in L2F application with the use of behavioral supports and (2) general performance on L2F application for each participant.

**Results:**

All three participants completed the initial auditory noise assessment (ANA) as well as 8 or more levels of the L2F application with varying accuracy levels. One participant completed the entire L2F program successfully. Several behavioral supports were used to facilitate participation; however, each individual demonstrated varied levels of engagement with the application.

**Conclusions:**

The L2F application may be a viable intervention tool to support audiovisual speech perception in persons with complex communication needs within a school-based setting. A review of behavioral supports and possible beneficial modifications to the L2F application for persons with complex communication needs are discussed.

Autism spectrum disorder (ASD) is a neurodevelopmental disorder marked by deficits in social communication and restricted and repetitive behaviors (American Psychiatric Association, 2022). The language skills of persons with ASD are diverse including those that demonstrate intact receptive and expressive language skills to more severe deficits in communication (Rice et al., 2005). Approximately 30% of children with ASD present little or no functional speech by nine years of age (Anderson et al., 2007). The term “complex communication needs” is often used to describe the communication skills for this particular group of individuals with ASD. Characteristics of complex communication needs include considerable difficulties with unprompted basic social-pragmatic routines, far greater understanding of single word lexical items (including nouns) than other lexical categories, difficulty relating past and future events, and preference for visual subject matter (photos videos, computer graphics, animation) (Schlosser & Sigafoos, 2002; Schlosser et al., 2019). Persons with complex communication needs often have limited long-term outcomes in education, independence, and quality of life (Howlin et al., 2000), yet language interventions for this particular group of individuals remain significantly understudied compared to individuals with ASD who present with higher levels of communication abilities (Tager-Flusberg & Kasari, 2013).

Language is of central importance in ASD and several theories have been proposed to explain why particular persons with ASD struggle to develop language skills. One candidate contributor for observed differences in language is poor modulation of gaze to faces of others (e.g., Hobson et al., 1988; Klin et al., 2002). Limited gaze to the face in early development, coupled with difficulties processing speech by the communicative partner, may negatively impact the development of foundational language skills, leading to language impairment. The term used to describe the simultaneous processing of visual and auditory speech cues during communication is audiovisual (AV) speech perception. This process involves the integration of what individuals see (mouth movements) with what they hear (auditory speech). The process of successfully developing AV speech perception is dependent on listeners being able to use both heard *and* seen speech to comprehend a speaker’s message. Indeed, we know that the visual information from a speaking face, particularly our ability to see a speaker’s mouth, can enhance our understanding of a spoken message, especially in the presence of background noise (e.g., Grant et al., 1998; Grant & Seitz, 2000; MacLeod & Summerfield, 1987; Sumby & Pollack, 1954).

While much research on individuals with ASD has focused on social aspects of communication, less work has investigated AV speech perception in this population (Irwin & Diblasi, 2017). The existing data on AV speech perception in children with ASD (aged 6–14) indicates deficits in AV speech perception when compared to their neurotypical peers (Iarocci et al., 2010; Irwin et al., 2011, 2023; Smith & Bennetto, 2007). Further, children with ASD demonstrate differences in scan patterns of the face when compared to others, while also not utilizing the visual speech information they do take in to the same degree as neurotypical peers (Irwin et al., 2011, 2022). Children with ASD may also demonstrate difficulties discriminating fine-grained differences between phonemic information (Irwin et al., 2023), which may contribute to cascading negative effects in the development of semantic and syntactic skills (Joanisse & Seidenburg, 2003).

Further, efficient AV speech perception can be an essential component to understanding speech in noise. Visual information during speech can provide critical cues when trying to understand speech in noise, leading to better processing of the intended message (Sumby & Pollack, 1954). In addition to demonstrating different gaze patterns to a face, adolescents with ASD have particular difficulty with listening in noise (Alcántara et al., 2004). When compared to typically-developing children, children with ASD look less at speaking faces overall and less to the speaker’s mouth, when auditory noise is present (Irwin & Brancazio, 2014; Irwin et al., 2011). Therefore, persons with ASD may be at a significant disadvantage in noisy environments such as classrooms where AV speech perception is necessary for effective listening and often tied to academic success.

Although previous research in AV speech perception in autism has focused on school-aged children, a few studies have investigated AV speech perception in adults with ASD (Keane et al., 2010; Pelphrey et al., 2002; Saalasi et al., 2012). Keane et al. (2010) reported no differences in the processing of congruent and incongruent AV information between adults with high-functioning ASD compared to neurotypical peers, whereas Saalasi et al. (2012) reported weak AV integration on a McGurk task for adults with ASD when compared to neurotypical peers. Further, Pelphrey et al. (2002) reported that adults with ASD do not attend to the key “T” elements of the faces (e.g., scanning from eye to eye and then down the center of the face to the mouth) as neurotypical adults do.

The aforementioned studies mainly investigated people with ASD who demonstrated high oral language abilities. Although many children with ASD develop language and communicate orally, a substantial number of persons with ASD present more complex communication needs. It is possible that deficits in AV speech perception are *even more* prominent in persons with ASD with greater language and cognitive needs and a higher propensity toward self-injurious or other maladaptive behaviors (Bal et al., 2016; Howlin et al., 2014; Lord et al., 2004). Indeed, researchers have established that global sensory processing deficits may be a key contributor to the unique variance present within the autistic population (Dellapiazza et al., 2020; Robertson & Baron-Cohen, 2017) and persons with more complex needs may demonstrate more significant sensory processing deficits. Therefore, intervention programs which strategically target the simultaneous processing of different sensory inputs may provide an innovative and efficacious way to improve language and listening skills for this particular population. Given the difficulties with studying persons with ASD who present more complex needs, fewer research studies have been conducted on intervention practices in this population. To the authors’ knowledge, to date, no intervention studies which focus on AV speech perception or listening within noise have been conducted with this group. These individuals are often considered the “neglected end” of the autism spectrum (Tager-Flusberg & Kasari, 2013). Thus, this is a critical area of study and this population should not be overlooked.

Given the scarcity of research on persons with complex communication needs and ASD, and the need for evidenced-based interventions, studies including this population are warranted. Therefore, the current feasibility study is designed to provide preliminary evidence on the use of a specifically designed iOS therapeutic training application called *Listening2Faces* (L2F). L2F is a theoretically driven, researcher-developed application designed for use with an iPad. The L2F application is an interactive, adaptive program that presents videos of speakers producing monosyllabic words in varying levels of auditory noise. The goal of the program is to improve listening skills by encouraging students to access/look at visual cues from the speaker's mouths to improve understanding of spoken single words in noise. Preliminary data of 8–10-year-old verbal children with ASD found that the use of this therapeutic training application increased their ability to understand speech in noise of familiar and unfamiliar speakers (Irwin et al., 2015). All of the children exhibited increased accuracy in identifying words in noise with the help of a speaking face. At the end of the training, all children progressed to the highest levels of the program where the level of background noise was so high that the spoken messages were barely audible.

Given the severity of language impairments in persons with complex communication needs, finding interventions that are engaging and interactive and that can be conducted with minimal support of an educator or paraprofessional, is difficult. The L2F application supports the improvement of AV speech perception using single-word spoken vocabulary. This level of language input is accessible to persons with complex communication needs and may be a vital ‘stepping stone’ in the intervention process in supporting improved AV speech perception skills. Therefore, the purpose of this study was to determine the feasibility of using this L2F application with young adults with ASD who present complex communication needs. To that end, we ask two main empirical questions: (1) What was the level of participation in the L2F application when provided with evidence-based behavioral supports within the school setting for young adults with complex communication needs? (2) What does the preliminary data collected by L2F within the school setting show for 3 young adults with ASD and complex communication needs?

## Method

### Participants

Three White students ages 17;11, 20;1, and 21;11 (2 males, 1 female) were recruited for participation through a private day school in Rhode Island. This day school provides specialized treatment and tailored educational services for students with ASD, intellectual disabilities, and other developmental disorders. Students at the school typically present with significant behavioral challenges and co-occurring diagnoses that cannot be safely managed in a typical school setting. The school focuses on positive psychology principles and the development of effective coping skills, while maintaining a behavioral intervention approach. Each participant in this study was previously diagnosed with an intellectual disability in the severe to profound range. The participants were not excluded for the presence of co-occurring psychiatric diagnoses, the use of medication, or the presence of seizure disorder.

The study was approved by the University of Rhode Island’s and the school’s Institutional Review Boards. Assents/consents were obtained from parents and students prior to the start of the study. A research assistant was trained on the L2F application and data collection procedures. This research assistant conducted each individual session with the participants as well as all cognitive behavioral interventions which took place prior to participation with the application. All sessions took place within the school setting during school hours.

### Procedures

A single group pretest–posttest design was employed to determine the effectiveness and acceptability of the L2F  application with a group of 3 young adults with complex communication needs. Prior to the start of the L2F session, all students participated in desensitization procedures (see below for details). A screening procedure was also performed to ensure that participants had familiarity with target words used in the application and were able to engage in a picture identification task with at least 80% accuracy, without exposure to noise and/or visual presentation of a speaker on the application. Forty words within the L2F application and their picture displays were randomly chosen for the screener and embedded into a PowerPoint presentation. The PowerPoint was presented on an iPad, similar to the one used for the L2F application. Participants were asked to point to the stated word when shown the picture within a 4-picture display. The students’ accuracy levels on the screener are as follows: Kim had 80% accuracy, Zane had 85% accuracy, and Jack had 98% accuracy.

Finally, the L2F application was designed to be implemented 24 min per day, 5 days per week for 4 weeks. Given (1) the complex needs of the current participants, (2) the classroom demands/curriculum requirements, and (3) availability of the research assistant and other staff within the school setting, this schedule was not adhered to for the following pilot study. Participants were encouraged to attend a L2F session 2–3 times weekly with the research assistant. This schedule was modified for bouts of illness, school vacations, and if/when participants were dysregulated and unable to safely attend sessions. The goal of each session was to complete one level, which included 3, 8-min blocks with breaks in between. Visual timers and positive feedback from the research assistant were provided to encourage the participant to keep working through a block; however, participants could refuse or stop at any time.

#### Cognitive Picture Rehearsal and Desensitization Routines

Cognitive picture rehearsal is an instructional strategy that can be used to reduce stress and anxiety in children with ASD as they may lack appropriate coping mechanisms (Groden et al., 2002). Picture rehearsal exposes a child repeatedly to a sequence of pictures in order to handle a particular situation using a script. The picture rehearsal scenes created for a child are based on the positive reinforcement principle of learning theory. This approach capitalizes on the strengths of children with ASD (Groden et al., 1988) by focusing on the visual system and providing structured interactive routines.

For our study, cognitive picture rehearsal provided a strategy for young adults with ASD to adjust to the new setting of the laboratory space where they would participate in the L2F sessions specifically. Cognitive picture rehearsal was included each day that a participant went to the laboratory space and typically required no more than 5 min to rehearse the scene using a script and pictures that displayed the laboratory. The research assistant read the script to the participant. Each participant heard the picture rehearsal once in its entirety and then was required to “repeat” or rehearse the scenes with the research assistant. Based on the communication abilities of the learner, the scenes were repeated verbatim, or the participant performed a cloze task where they “filled in the blanks” by repeating key words when prompted by the research assistant. The goal of cognitive picture rehearsal was for participants to imagine practicing the desired behavior successfully, not to memorize the words. After the second reading, the research assistant asked questions to gauge comprehension of the scene. Furthermore, desensitization procedures for this study were designed based on cognitive picture rehearsal, visits to the laboratory space, and conversation with the research assistant as to what the application entails.

#### Preparation and Desensitization Using Cognitive Picture Rehearsal

Participants were exposed to the laboratory space (a designated room for research within the school) four times prior to starting the study. These visits coincided with the word screening. Procedures for exposure/desensitization to the laboratory are as follows for each individual visit:

**Fig. 1:**
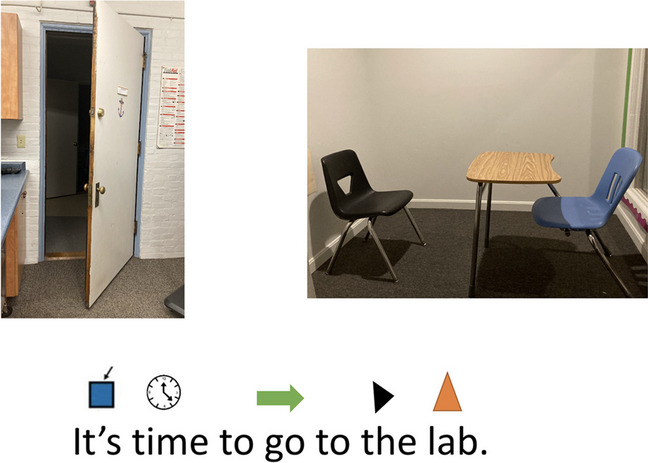
“Going to the Lab” cognitive picture rehearsal scene

1. The student reviewed cognitive picture rehearsal scenes for relaxation “Going to the Lab” (Fig. 1) with a research assistant.
2. The student walked to the laboratory space with a staff person.
3. The student chose an item from the “prize box.” If they chose an edible prize, they were given time to eat the item. If they selected a tangible prize, they were given 3 min to play with the item.
4. The student earned reinforcement based on their individualized behavior plan before transitioning back to the classroom with staff.

The research assistant prepared the materials and several procedural steps to follow in order to engage with the students and support their use of the L2F application. First, the research assistant calibrated the application prior to the student entering the room. The research assistant then presented the iPad (with the L2F application) and said, “time to play the game.” Subsequently, if the student did not respond within 20 s, the application paused and an “are you there?” icon appeared. If the student continues to not respond, the research assistant provided the prompt “take your best guess.”

#### Listening2Faces (L2F) Application

**Fig. 2:**
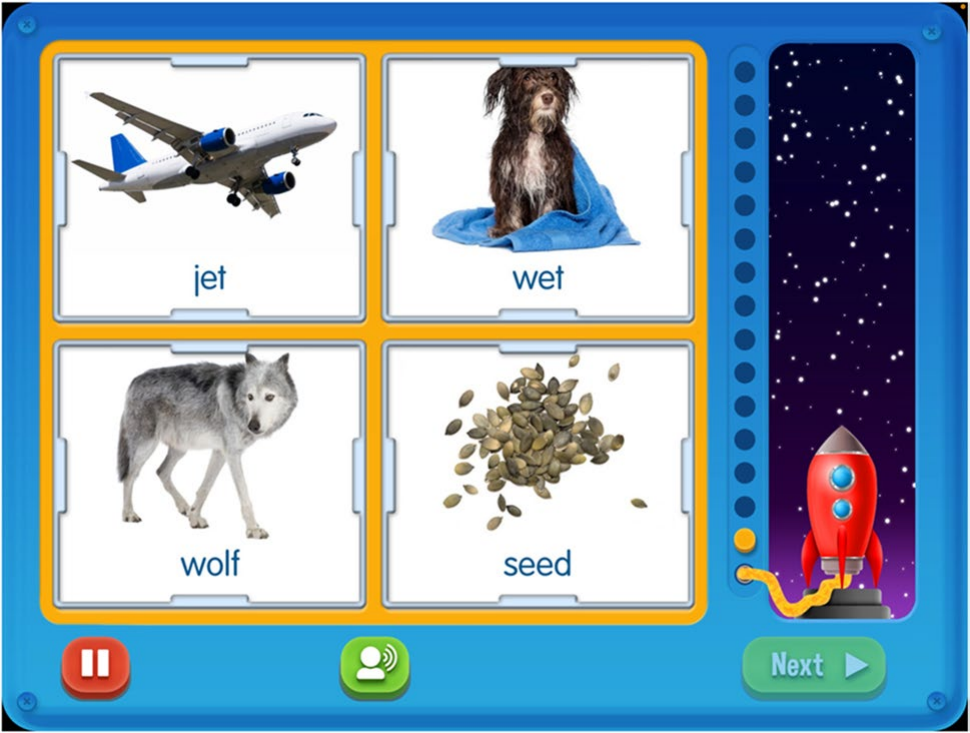
Example ANA trial

The L2F application includes an auditory noise assessment (ANA) administered at the beginning and end of the program to provide a sensitive and dynamic measure of the signal to noise ratio (SNR) for each child at each training session. SNR measures the strength of a signal compared to the strength of background noise and is measured in decibels (dB). A positive SNR indicates that the signal is louder than noise, and a negative SNR indicates that the noise is louder than the signal. The initial ANA calculates the environmental noise where the application is being used and the noise level chosen by the user in order to provide a SNR. Testing does not begin until the environmental noise is 50 dB or less. Then, the application allows the participant to choose a comfortable listening sound level for the words that are presented. This is an important feature of the application, for it allows the measurement of background noise in the environment to establish acceptable background noise levels and allows for the application to calculate the background noise levels for each training session. The ANA also provides a means to capture listening in noise performance (without the support of a speaking face) before and after completing the training protocol (i.e., completion of all 20 levels of the application). Fifteen words are presented auditorily, and the participant is asked to touch one of the four pictures that match the word they heard. Figure 2 depicts an example ANA trial.

The L2F program is an adaptive and interactive program that presents videos of speakers producing imageable monosyllabic words at varying levels of auditory noise. The application adapts to the participant’s performance by increasing in difficulty as performance improves. The speakers within the application vary in age, gender, and ethnicity as increased variability in the speech signal may support generalizability in perceptual training (Bradlow & Pisoni, 1999; Lively et al., 1993; Magnuson & Nusbaum, 2007; Rvachew, 1994).

**Fig. 3:**
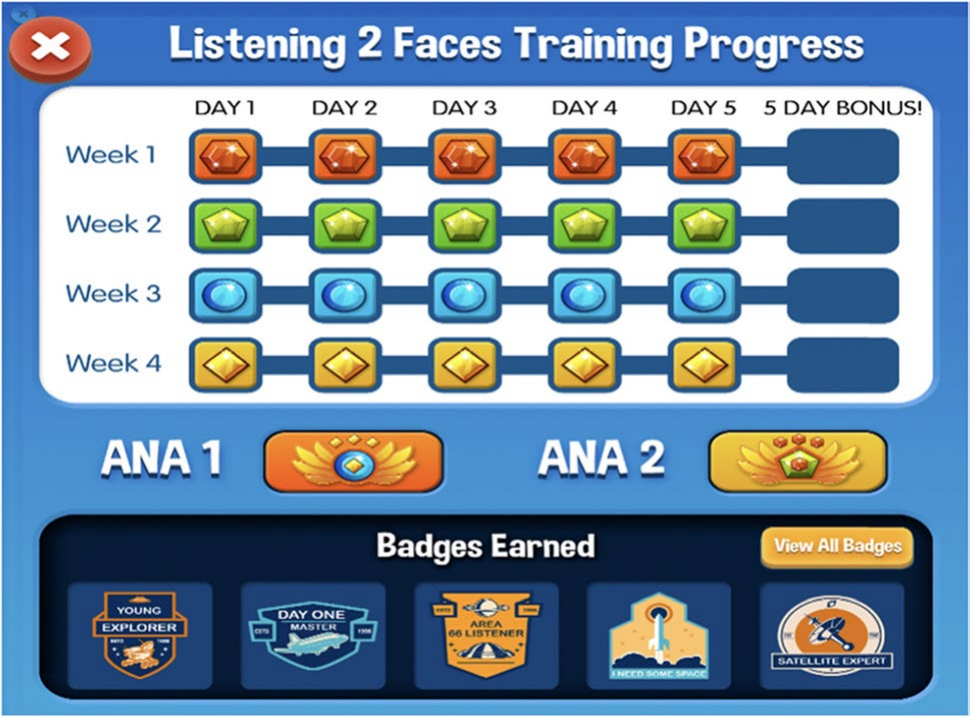
Example of gems and badges earned

**Fig. 4:**
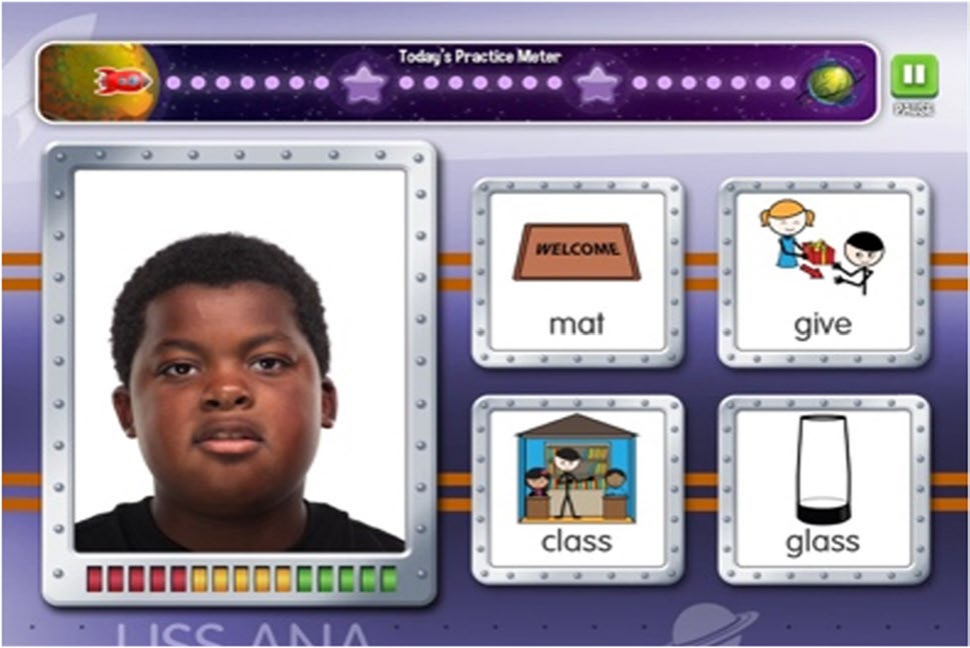
Example trial with speaker and images

The L2F application was created using a child-friendly space theme and includes game-like features to increase motivation and compliance. Users are prompted to help understand a speaker’s message as it is noisy because the speaker comes from far away and they are on a mission to help receive messages. Through each level (20 levels total), application users journey from planet to planet to earn gems and badges (see Fig. 3). Each level contains 3 blocks lasting for 8 min each. On the completion of each block, participants can choose to play a short reinforcing game. For each trial, a word is presented auditorily along with four images of words presented on the screen (one of the images matched the target word while the other 3 are similar words, see Fig. 4 for an example). Participants are presented a video clip of the speaker producing a word and is shown a set of 4 attendant illustrated images: a target word (e.g., glass), a matching onset (e.g., give), a matching rime (e.g., class), and a distractor (e.g., mat). The participant is asked to touch the word that they heard on the iPad to make their selection on the touch screen.

Noise levels within the application vary from participant to participant as it is adaptive in nature. Therefore, based on performance, the application will increase the noise level making it harder for a participant to hear the spoken word, and more reliant on visual information from the speaking face to support interpretation of speech. The adaptive nature of the noise was designed to settle on a noise level that is challenging but not impossible for each participant.

During a block, the program selects words and then it selects a speaker. The speaker-word combinations are randomized within a block. All stimuli are presented at 70 dB while the noise varies adaptively ranging from a SNR of + 40 to − 12 dB. When a participant meets a particular threshold of accuracy, the noise level increases for the next training block, and the size of the noise level increases in relation to the participant’s accuracy level. For example, if a participant correctly identifies at least 75% of the words, the noise level increases by 30, 20, 10, and 5 dB on subsequent blocks with at least 75% accuracy. When accuracy is between 45 and 75%, the noise level increases by 2 dB on the next block. However, when accuracy is below 45%, the noise level for the next block decreases by 5 dB.

### Measures

All students completed the *Autism Diagnostic Observation Schedule-Second Edition* (ADOS-2; Lord et al., 2012) with the second author and met criteria for an ASD diagnosis. The ADOS-2 is a semi-structured, standardized assessment of communication, play, social interaction, and restricted and repetitive behaviors. The ADOS-2 is the gold standard clinical tool for the observational assessment and diagnosis of ASD and provides a comprehensive picture of a person’s current symptomatology.

The *Receptive One Word Picture Vocabulary Test 4th Edition* (ROWPVT-4; Martin et al., 2011) was administered for descriptive purposes and results are included in Table 1. The ROWPVT-4 is an assessment tool that measures receptive single word vocabulary. Examinees are asked to point to pictures corresponding to a spoken word. The ROWPVT-4 is a standardized assessment with a mean of 100 and standard deviation of 15. Although all participants were able to perform the picture identification protocol by independently pointing to a spoken work produced by the examiner, they all exhibited very low receptive vocabulary with raw scores ranging from 64 to 90 and all standard scores falling below 55.

**Table 1:** Participant demographics

Participant	Age (year; months)	ADOS-2 Module	ADOS-2 Composite Score	Classification	Diagnosis	ROWPVT-4 Raw Score	ROWPVT-4 Standard Score
Kim	17;11	3	10	Autism	Autism High	64	< 55
Zane	20;1	2	10	Autism	Autism High	77	< 55
Jack	21;11	2	10	Autism	Autism High	90	< 55

*Participant names have been changed to preserve anonymity

As seen in Table 1, each of the participants obtained a classification of “autism” and diagnosis of “High” on the ADOS-2. However, it should be noted that the verbal abilities of the participants varied greatly. For example, Kim was administered the ADOS-2, module 3, given her ability to produce connected oral sentences. Despite significant deficits in receptive language, Kim was able to produce simple and more complex sentence patterns. She produced the following sentences during the ADOS-2 administration: “She is swimming in the pool and he is swimming in the pool. I love to pretend at school. I want to play with the playdough.” Kim’s prosody was atypical, including odd intonation patterns; however, there was evidence of more complex grammatical structures within her speech, including use of conjunctions and pronouns. Zane and Jack were administered the ADOS-2, module 2, due to less verbal skill. Zane produced short (2–3 word) utterances during most interactions; however, when provided with the ADOS-2 storybook task, he generated the sentence “The frogs are falling in the water.” Jack produced no verbal language during the administration of the ADOS-2; however, classroom staff reported that he produces single words at times. Jack was able to understand simple single word spoken vocabulary as evidenced by the ROWPVT-4 and to follow simple directions during the administration of the ADOS-2. Classroom staff reported that Jack follows simple directions in the context of the school day. Although the verbal abilities of the 3 participants varied greatly, all three demonstrated significant deficits in receptive language skills. The varied verbal abilities represented within the three participants is a nuanced representation of the heterogenous language abilities of a person’s ASD presenting complex communication needs.

### Data Analyses

Descriptive statistics will be used to characterize performance from the participants. Per participant, these data include overall time engaged with the L2F application, percent accuracy per level, percent accuracy per SNR, and performance accuracy by speaker age and gender. Baseline measures for level of participation included determining the ratio of participation/compliance in L2F within the first 3 attempts to have the student engage in a session (i.e., number of sessions attended/number of attempted sessions). This was then compared to the number of sessions attended out of the number of attempted sessions for the last 3 sessions.

## Results

### Level of Participation

The first empirical question sought to investigate the level of participation in the L2F application when modifications and accommodations were provided for persons with complex communication needs. Modifications were individualized for each participant (names have been changed to maintain anonymity) to support their participation in the L2F application, although these were met with varying success. All participants demonstrated 100% participation (attended 3/3 sessions) at baseline. This was then compared to the last 3 attempts for participation. Both Jack and Zane attended their last 3 sessions when invited (both participants demonstrated 100% participation in the application); however, Kim completed 0/3 of the last invited sessions. Kim demonstrated a significant decrease in participation following the first 2 weeks of participation. Please see below for a detailed description of participation for each student.

1. Kim: There were a total of 50 sessions where Kim was invited to participate in the L2F application. Kim completed a total of 13 sessions where she engaged with L2F. She refused to transition to the laboratory on 32 occasions and on 5 occasions, she transitioned to the laboratory with the research assistant; however, refused to use the L2F application. On average, Kim completed 1–2 blocks per session. Kim had a cognitive picture rehearsal ‘Going to the Lab’ that initially was beneficial in supporting the transition from the regular classroom setting to the laboratory space (in a different part of the school). Based on her interests, her incentive for participation was listening to music of her choice on an iPad, dancing in the lab, playing with a puzzle, or playing with another game for 5 min. However, the strengths of these reinforcing activities appeared to fade over time and Kim failed to consistently comply to go to the laboratory space and/or refused to engage in the L2F application once she came to the laboratory space. After several attempts over several weeks to encourage and motivate her to play the L2F application, Kim’s participation in the study was terminated.
2. Zane: There were a total of 30 sessions where Zane was invited to participate in the L2F application. Zane willingly transitioned to the lab space on all 30 occasions and participated in the application during each session. On average, Zane completed 1–2 blocks per session. Zane also participated in cognitive picture rehearsal that included scenes of “Going to the Lab,” “Lab Room,” and “Playing the Game” for his incentive. He independently worked on the application when in the laboratory space. Zane’s incentive was either a puzzle or Legos for 5 min following participation in the application. The prompt of “we can play with Legos after” was typically provided to support Zane’s continued participation in the L2F application. He was also very motivated by the application’s spaceship theme and visual schedule which depicted a spaceship moving from planet to planet on the iPad screen when advancing a level. Following completion of a session, Zane would often not engage in Legos, despite that being his incentive of choice, and head immediately back to the classroom. This structure and individualized modifications were helpful for Zane as he was able to complete the entire L2F program.
3. Jack: There were a total of 8 sessions where Jack was invited to participate. During each session, Jack was able to complete an entire level. His cognitive picture rehearsal included scenes of “Going to the Lab,” “Lab Room,” and “Playing the Game” for his prize. He was able to attend well and was successful in completing a level each time he came to the laboratory space. Jack’s participation began near the end of the academic school year. Although the research team anticipated continuing to work with Jack following graduation, this was unable to occur. Therefore, Jack did not complete the entire 20 levels of L2F application due to time constraints.

### Preliminary Outcomes

The second research question focused on the preliminary data collected by the L2F program for three young adults with ASD and complex communication needs. For each participant, the initial ANA scores are included (see Table 2 for further details), the number of sessions and minutes is shown, and the overall accuracies by level and by signal to noise ratio (SNR) are included.

**Fig. 5:**
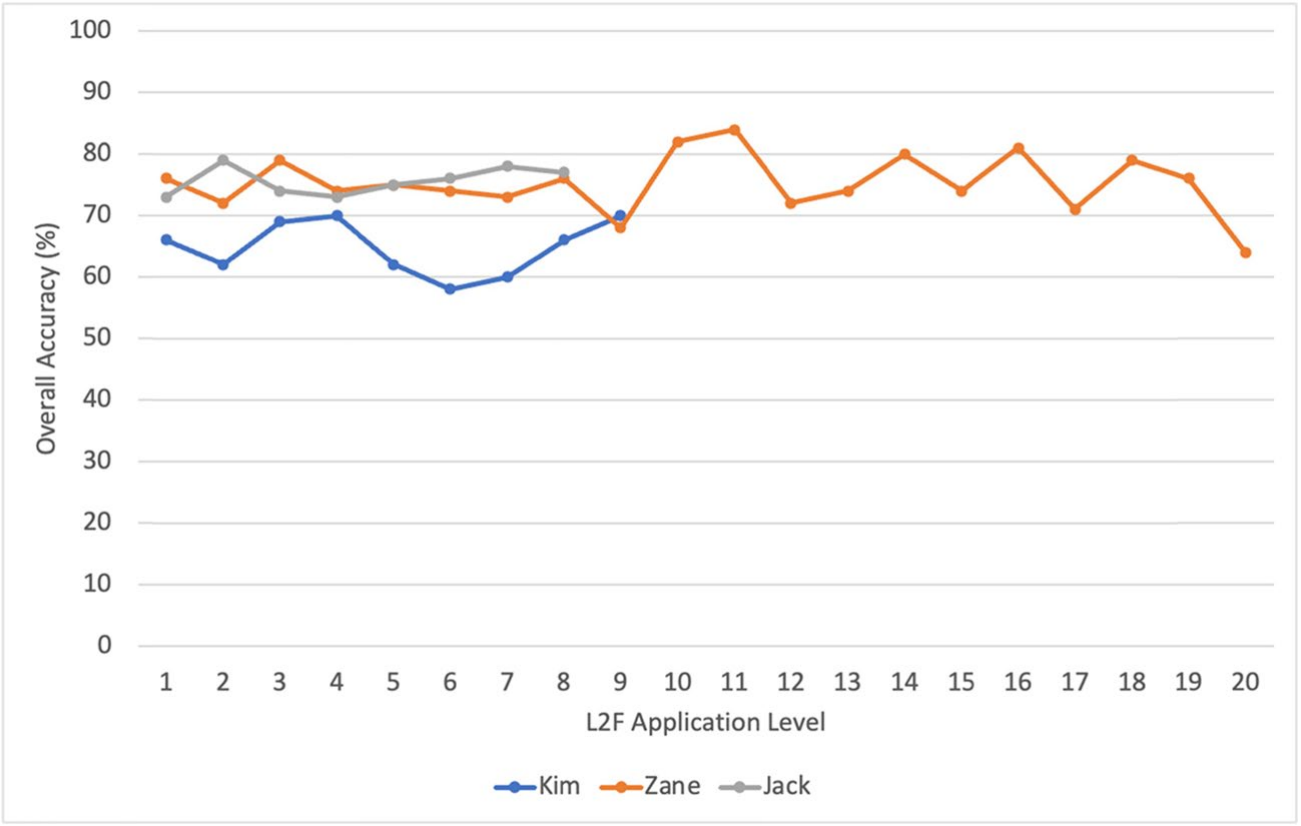
Accuracy scores for each level by participant

1. Kim: For the initial ANA, Kim obtained an accuracy score of 93% accuracy in selecting target words with a 0 dB SNR and a selected sound level of 72 dB. Within 15 sessions over 7 months, Kim completed 200 min (9 levels) before she was unwilling to continue participating. Kim participated on average for 13 min per session (ranging from < 1 to 24 min). For each level that Kim completed, her overall performance accuracy ranged from 58 to 70%.
2. Zane: For the initial ANA, Zane obtained an accuracy score of 93% accuracy in selecting target words with a − 5 dB SNR and a selected sound level of 61 dB. Zane completed 480 min (all 20 levels) of the L2F program over 30 sessions within a 7-month period. Zane was able to stay on the application for an average of 16 min per session (ranging from 2 to 24 min). For each level that Zane completed, his performance accuracy ranged from 64 to 84%. For the post-training ANA, Zane obtained an accuracy score of 93% in selecting target words with a − 5 dB SNR and a selected sound level of 72 dB.
3. Jack: For the initial ANA, Jack obtained an accuracy score of 66% accuracy in selecting target words within a SNR of − 5 and a selected sound level of 70 dB. Jack completed 192 min (8 levels) of the L2F program in 8 sessions within a 3-week period. Jack was able to stay on the application for 24 min for each session. For each level he completed, Jack’s performance accuracy ranged from 73 to 79%. Please see Fig. 5 for level-by-level accuracy scores for each participant.

**Table 2:** ANA scores for each participant

	Initial ANA scores				Post-training ANA scores			
	% accuracy	SNR	Ambient sound level	Selected sound level	% accuracy	SNR	Ambient sound level	Selected sound level
Kim	93%	0 dB	39 dB	72 dB				
Zane	93%	− 5 dB	46 dB	61 dB	93%	− 5 dB	43 dB	72 dB
Jack	66%	− 5 dB	45 dB	70 dB

#### Signal to Noise Ratio (SNR)

**Fig. 6:**
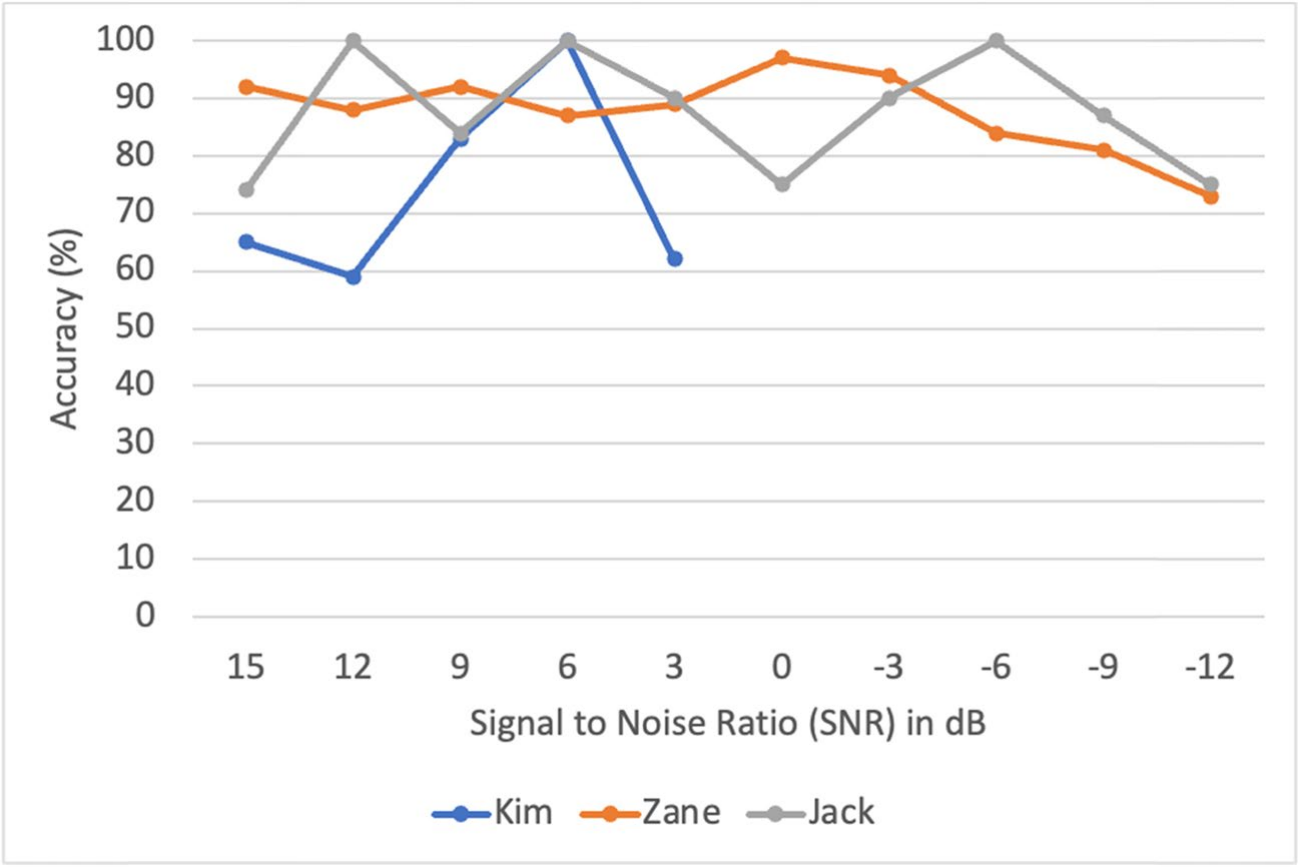
Performance accuracy by signal to noise ratio (SNR)

Additional data collected by the L2F program included participant’s performance accuracy broken down by SNR ranging from + 15 dB, where the signal is stronger than the background noise, to − 12 dB, where the noise is stronger than the signal. Figure 6 demonstrates each participant’s performance accuracy based on the SNR ranging from easiest listening environment (+ 15 dB, far left) to the hardest/noisiest listening environment (− 12 dB, far right).

As demonstrated by the data, Kim did not progress to a point in the application where noise was louder than the speech signal (0 dB SNR). However, both Zane and Jack demonstrated high levels of accuracy in negative SNR trials.

#### Speaker’s Age and Gender

**Fig. 7:**
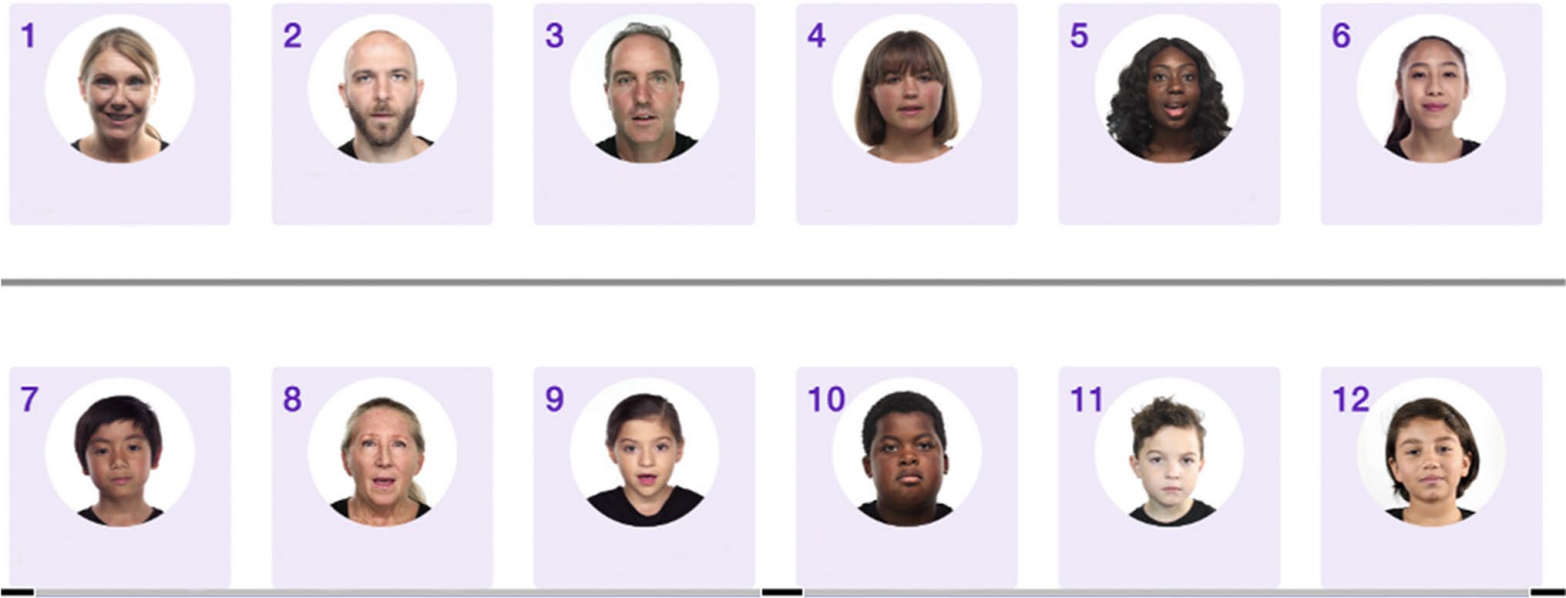
Example images of speakers

The L2F application also includes data of the speaker’s age and gender. Figure 7 shows example images of some of the speakers within the L2F application. Table 3 includes each participant's performance accuracy based on the speaker’s age and gender. At the bottom of Table 3, performance accuracy has also been aggregated across (1) all male speakers and (2) all female speakers. Participants showed no accuracy differences between the genders of the speakers.

**Table 3:** Performance accuracy by speaker’s age and gender

Speaker’s age	Speaker’s gender	Kim’s percent accuracy	Zane’s percent accuracy	Jack’s percent accuracy
6–8	Male	68	73	79
6–8	Male	61	81	74
6–8	Female	61	75	78
9–16	Female	74	71	72
9–16	Female	60	70	74
9–16	Male	65	63	61
9–16	Female	64	88	72
16–24	Male	80	79	79
16–24	Female	65	78	77
24 +	Male	60	74	79
24 +	Male	66	81	78
24 +	Female	68	77	77
24 +	Female	57	73	71
	All male speakers	65	75	76
	All female speakers	65	75	75

## Discussion

Audiovisual (AV) speech perception is a fundamental part of language development. The purpose of this study was to investigate the feasibility of using the Listening2Faces (L2F) application with persons with ASD who demonstrate complex communication needs. L2F application is designed to improve listening to speech in noise by urging users to utilize visual cues gleaned from the speaker’s mouth which in turn facilitates comprehension of individual words in the presence of background noise. This unique design promotes a scaffolded approach in promoting the simultaneous integration of both visual and heard speech. This simultaneous integration of different sensory inputs may be particularly difficult for persons with ASD. Previous research has documented decreased AV speech perception skills in the ASD population (Irwin & Brancazio, 2014; Irwin et al., 2011). Given the limited research on persons with ASD with complex communication needs as well as the specific design of the L2F application, here, we sought to determine whether L2F has the potential to support AV speech perception in this understudied population. Persons with complex communication needs have difficulty with interpreting connected speech and more complex grammatical structures. As the L2F application focuses on speech in noise at the single word level (which is a relative strength for this population), this application has the potential to be used independently with persons with complex communication needs. Therefore, the current investigation was designed as an exploratory approach to collect preliminary data on the use of the L2F application with a small cohort of 3 young adult participants with ASD and complex communication needs. Two research questions were posed: (1) when provided with evidence-based behavioral supports, what was the level of participation in the L2F application for persons with complex communication needs and (2) what did the preliminary data collected within the L2F application indicate.

In regard to the first research question about level of participation, individual modifications were necessary for each participant. Cognitive picture rehearsal scenes appeared beneficial in supporting the successful transition between the classroom setting and the laboratory space as most participants willingly attended training sessions with the research assistant. However, although Kim initially transitioned easily with the research assistant, she later became unwilling to participate in sessions. Further, Kim and Zane consistently used cognitive picture rehearsal every time they transitioned to the laboratory space while Jack readily came with the research assistant to each training session upon seeing her.

Additionally, tailored incentives, based on participant preferences, were used to ensure motivation, persistence, and compliance within L2F training sessions. All participants were aware of their incentives and were able to request them upon completion of individual blocks. However, there were variations among participants as to the consistency of reinforcers which followed completed blocks. Initially, Kim was motivated to attend training sessions with the research assistant. She persisted in completing several blocks within a single training session and was motivated by incentives such as music or time for dancing. However, after 15 sessions, Kim refused to attend training sessions, despite reminders of incentives and use of cognitive picture rehearsal. There were 5 occasions where she transitioned to the laboratory space and engaged with the RA in an activity; however, she refused to participate in the L2F application. On the other hand, Zane wanted to know that Legos was an incentive he could obtain, but after using the L2F application, he would return to class without playing with the Legos. Lastly, few incentives were used for Jack to complete levels. Once attending a session, visual timers and verbal prompts were used to support his completion of blocks. Therefore, although he did not complete all the levels due to graduation from his school program, he appeared to have the capacity to complete the entire L2F application given his attention and engagement displayed during the completed sessions.

When collectively considering the variations in responses to cognitive picture rehearsal and incentives, the patterns of behaviors exhibited by the participants are representative of persons with ASD presenting complex communication needs, as students may quickly satiate and reinforcers change over time (Prior et al., 2023; Sigafoos & Gevarter, 2019). Although preferred reinforcers were used with each student based on their current classroom programs, in the future, conducting a reinforcer assessment may prove to be helpful for L2F users who require behavioral support. There are also standardized reinforcements already built into the application, such as earning of badges and a visual schedule to track progress; however, not all participants, especially those with complex communication needs, may be motivated by the reinforcements within the application. Additionally, the current space theme was reinforcing for one of our participants, but did not appear reinforcing for all. The application may benefit from an array of themes that a student would be able to choose based on their interest.

There are several considerations that should be made in order to modify the L2F application itself to be more user-friendly for persons with ASD with complex communication needs. As previously mentioned, each level was created to be completed in 24 min in a day. It is possible that shorter levels and shorter sessions for some students would be beneficial. In regards to the application’s interface, it would have been helpful to stop the program whenever a participant needed a break. Furthermore, the ability to repeat the audio would be beneficial for students as they may have been distracted when the audio played the first time. In regard to the noise included in the application, white noise may be bothersome to some students more than others. Therefore, an option to change the type of noise (pink or brown noise) presented in the application would be a beneficial modification. Additionally, if there is already noise in the environment, it may be helpful to add the level of noise required for the block or level rather than requiring a quiet space for the application to be used.

Lastly, students with complex communication needs may vary in the amount of support they may require from another person while using the application. Some participants may be independent and can use the application on their own, while others may need verbal reminders, physical prompts, or reinforcers to stay motivated and on task when using the application. If an individual does require additional support, the support staff should receive basic training so as to not intervene in the training sessions, which would invalidate results. Support staff are encouraged to motivate the student to persevere through the application. Additional training on the application itself may be helpful for the support staff to feel comfortable with the technology and troubleshoot difficulties as they arise. One of the participants in the study discovered a “workaround” on the application and was able to find a pause button. During these types of instances, it is worthwhile to have an assistant or paraprofessional involved so that they can help the student continue using the application as intended.

The second research question focused on the investigation of the preliminary data collected by the L2F program for three young adults with ASD and complex communication needs. Kim obtained an initial ANA of 93%, unfortunately, she did not complete the study and therefore her post-ANA could not be analyzed. As seen by her progress represented in Fig. 6, Kim’s accuracy levels stayed above 60% with increasing SNR; however, she never progressed to the point in the training sessions where the noise was greater than the “signal” (in this case speech). Jack’s initial ANA was 66% accuracy. Jack demonstrated a high level of participation in the program, completing 1 full level (24 total minutes) for each session he attended. When considering his progress, Jack appeared to maintain a high level of accuracy (above 70%) with an increasing SNR (see Fig. 6), suggesting that he may be “improving” listening within noise during the training sessions. Jack maintained a 70% accuracy level at − 12 SNR, meaning he was able to accurately choose target words when there were high levels of background noise. However, Jack also did not complete the study due to his school graduation. This is unfortunate, given his progress, as Jack had the potential to demonstrate significant differences in his post-ANA given his progress during his sessions.

Finally, Zane obtained a pre-ANA score of 93% accuracy and a post-ANA score of 93%. Although there were not significant differences in pretest to posttest data, Zane appeared to maintain a high level of accuracy (above 80%) when greater levels of noise were introduced at each level. For example, when considering Fig. 6, at a − 9 SNR, Zane maintained an accuracy level of ~ 80%. This suggests that greater noise than signal (i.e., speech) was present in the training, yet Zane maintained a high level of accuracy. It is possible that he was utilizing visual cues from the speaker’s face within the training levels to maintain that accuracy. This may be a critical feature of L2F that specifically can support students who do not typically seek out information from a speaking face. For students with complex communication needs who often avoid looking at speaking faces, this unique design can support the gaze shift to the face and the use of the visual cues “as a necessity” in order to successfully identify the target word. This simultaneous act of listening to auditory speech and looking at visual speech within the context of the game-like application may be an initial step in promoting improved looking to speaking faces. When considering the level-by-level accuracy data, each student demonstrated the ability to complete several levels of the program with accuracy levels above 60%. Although accessing speech in noise is difficult, these participants were able to do so. Even in the presence of noise (up to − 12 dB SNR), participants were able to use visual cues provided by the speaker to be at an accuracy level above chance. These accuracy levels remained relatively consistent among speakers of different ages and ethnicities. There were no differences in accuracy level related to gender across all three participants showing that the ability to access speech in noise did not vary by gender. Overall, the data suggests that mechanisms which cue visual speech information through the L2F application may facilitate perception of speech in noise for persons with complex communication needs.

### Limitations and Future Research

This feasibility study has several limitations. First, only three participants were included in this study and only one of the participants completed all 20 levels of the L2F application. There is significant variability among persons with complex communication needs, and thus, further research needs to be conducted with more participants within this population. Additionally, although the literature documents AV speech perception difficulties in the ASD population, to our knowledge, no research has specifically investigated AV speech perception in persons with complex communication needs. Therefore, the degree to which AV speech perception was impaired in these participants prior to participation in the study is unknown.

Furthermore, a prescribed schedule was not followed for the participants. Initially, the application was intended to be used for 24 min per day, 5 days per week for 4 weeks (Irwin et al., 2015). Due to the complex needs of the participants and restraints with conducting research within a school-based setting, a more flexible schedule was implemented. Future research may consider a more prescribed schedule and investigate if persons with complex communication needs are able to continue with the application using a more rigorous and frequent schedule. Finally, the current participants were young adults with complex communication needs. Young adults may be at a more “stable” part of their language learning trajectory compared to younger students whose language learning capabilities may be more susceptible to change. This study is a first step in investigating whether persons with complex communication needs can participate and use an application that focuses on speech in noise. In the future, it would be beneficial to recruit younger children with complex communication needs to explore if they are also able to use the application and perhaps improve their ability to understand speech in noise.

Future research should consider continuing to examine how and if persons with complex communication needs process and tolerate noisy speech. It is possible that persons with complex communication needs that have AV speech perception deficits could benefit from AV interventions and thus would be an area of future research.

## Data Availability

The data for this project is maintained at the University of Rhode Island in a secured database. The dataset analyzed for the current study is available from the corresponding author on reasonable request.
